# Exploiting Mobile Gamification to Foster Physical Activity: A Remotely-Managed Field Study

**DOI:** 10.3390/s23052598

**Published:** 2023-02-26

**Authors:** Giorgio Olivas Martinez, Valeria Orso, Alice Bettelli, Luciano Gamberini

**Affiliations:** 1 Department of General Psychology, University of Padua, 35131 Padua, Italy; 2 Human Inspired Technologies Research Centre, University of Padua, 35121 Padua, Italy

**Keywords:** physical activity, gamification, mobile method, field study, fitness app, data log

## Abstract

Physical inactivity is a plague for public health, especially in Western Countries. Among the countermeasures, mobile applications promoting physical activity seem particularly promising, thanks to the spread and adoption of mobile devices. However, the dropout rates of users are high, thereby calling for strategies to increase retention rates. Moreover, user testing can be problematic, because it is typically conducted in a laboratory, leading to a limited ecological validity. In the present research, we developed a custom mobile app to promote physical activity. Three versions of the app were implemented, each featuring a different pattern of gamification elements. Moreover, the app was designed to work as a self-managed experimental platform. A remote field study was conducted to investigate the effectiveness of the different versions of the app. Behavioral log data of physical activity and interaction with the app were collected. Our results show the feasibility of using a mobile app running on personal devices as an independently managed experimental platform. Moreover, we found that gamification elements per se do not ensure higher retention rates, rather it emerged that the richer combination of gamified elements was effective.

## 1. Introduction

Physical inactivity is the fourth leading cause of death worldwide [1]. Indeed, 5.3 million people die from non-communicable diseases yearly due to physical inactivity, including breast and colon cancers, type 2 diabetes, and coronary heart disease [2]. The World Health Organization is committed to reduce the prevalence of physical inactivity by 10% by the end of 2025 [3], drawing even more attention to this phenomenon.

The restraint measures, widely taken to contrast the spread of COVID-19, have further reduced the general level of physical activity, mainly because citizens were forced to stay in their homes [4,5]. Besides, lockdowns and other travel-limiting measures have restricted access to gyms, parks, and other places where people can train and work out [6]. On the flip side, home confinement and increased time availability have fostered the use of digital communication technologies [7]. Therefore, even though the pandemic caused a further decrease in physical activity, the adoption of mobile health-related applications (mHealth) has increased, thereby opening up new perspectives for promoting physical activity.

This trend indicates the potential to reach a large number of individuals with smartphone-based interventions promoting and supporting physical activity at a relatively little cost [7]. Notably, mobile methods allow to remotely collect data of actual app usage in a non-invasive manner and in a fully ecological context [8].

A growing body of evidence indicates that mHealth can positively influence behavior change, resulting in improved health outcomes. However, for what specifically concerns physical activity, the results are mixed [9]. This may be due to the poor quality of the apps or the limited inclusion of gamification and competition elements [9]. Yet, the potential of gamified mobile apps to promote higher levels of physical activity is well exemplified by the Pokémon GO app, which has been able to increase the number of young adults who reach 10,000 steps per day [10].

The study presented in this paper has the explicit purpose of helping people become more physically active, leveraging gamification to improve engagement and promote behavior change. Additionally, in order to face the new challenges presented by the pandemic and isolation situation, it exploits mobile methods to achieve a no-contact and fully remote field study. To do this, we purposefully developed a mobile app promoting physical activity designed to be the experimental platform in which the participant should conduct all the experimental phases. Moreover, to evaluate the effectiveness of game design elements, three app versions that differ in the richness of gamification features implemented were developed. Participants were randomly divided into three groups, each associated with a different experimental condition running on a dedicated version of the app.

We collected data logs about app retention time on the user’s device, behavioral data regarding the number of workout sessions, and user performance, to investigate the effect of different patterns of game elements. The rest of the paper is organized as follows. The next section will present the related works. Subsequently, the study will be described, focusing on the developed app, the experimental method, and the procedure. Finally, the analyses will be detailed and the outcomes discussed.

### 1.1. Gamification to Foster Behavior Change

In the past years, numerous apps have been developed to help users increase physical activity both through daily monitoring (e.g., step counters) and through performance-related push notifications [11]. However, up to 75% of users who install health apps quit using them within two weeks from the first installation [12].

To overcome these high dropout rates, a number of different strategies have been experimented. The presence of social elements, for example, has proven to be an effective motivational leverage for gym fanatics, leading many fitness enthusiasts to spend several hours each day in intensive training sessions for the only purpose of being able to “post” successful selfies on their Instagram accounts [13].

In the same vein, many apps have embedded elements typical of the world of video games, such as badges and levels, managing to increase the engagement of their users [14]. Notably, the apps that decided to remove these elements, considering them superfluous, such as Nike+, which removed the badges from its apps in 2016, found a reduction in involvement, generating a sense of dissatisfaction among its users [15].

Many researchers have emphasized the importance of investigating the role of gamification in apps designed to promote physical activity [16]. In particular, Johnson and collaborators [17] found that gamified smartphone apps are in fact capable of increasing the level of physical activity.

In the present work, we refer to the definition of gamification proposed by Deterding, who conceptualized it as the “use of game design elements in non-game contexts” [18]. More specifically, with game design elements, we refer to features that, once introduced in non-playful contexts, can replicate playful dynamics without necessarily turning the application into an actual game. There are many different possible game elements, but points, ranking, and badges are the ones most commonly employed [19]. Often these elements are simply added to the application without taking into consideration the user experience or the generated dynamics, resulting in a losing approach known as pointification [20]. In recent years, however, other approaches have been developed that try to incorporate more aspects of the experience, such as the Smart Gamification Model by Kim [21]. Her model emphasizes the importance of applying different mechanics depending on the user’s specific needs and expertise level [21]. More specifically, she breaks the player journey into three stages, each identifying a different type of player. In particular, newbies need onboarding elements to understand and appreciate the new game world into which they are introduced; regular players are already familiar with the game mechanics and require habit-building elements to master it. Finally, player enthusiasts need elements to recognize their ability after reaching total mastery of the game.

### 1.2. Leveraging Mobile Apps to Run Field Experiments

The distinctive feature of field experiments is that they are conducted in natural environments. Their main value is thus attributed to the high ecological validity, because it increases the researcher’s confidence that the phenomenon under investigation naturally occurs and follows its spontaneous unfolding. With that respect, the emerging field of mobile methods is particularly promising because it enables the collection of detailed event logs of naturally occurring behaviors by leveraging mobile communication technologies [8].

Furthermore, there are many additional advantages of using mobile apps as data collection tools. Firstly, scaling, which is the ability to reach a large pool of participants with relatively few resources, is a common struggle in field experiments [22]. With that respect, virtually everybody owns a smartphone and/or a tablet [23]. In the United States 95% of adults aged 18–35 years and 60% of adults aged over 50 years own a smartphone [24]. This wide penetration potentially makes every device an intervention facilitator and a data collection tool to track thousands of people interacting with the platform [22].

A further benefit concerns the control over the randomization and the delivery of the experimental materials. In this regard, mobile apps can automate the random assignment and control material delivery, avoiding human errors and ensuring strict double-blind experiments [22]. According to Helbing and Pounaras [25], mobile methods could become the new gold standard for accurate measures of real-time behavior changes.

Still, we have to acknowledge that mobile methods have an important limitation amounting to the generalizability of the results to the entire population. Indeed, the sample is inherently biased, because participation in the study usually requires a certain level of expertise and familiarity with these technologies, thereby failing to represent digital illiterates. In the research of Rothschild and colleagues [26], for example, only 6% of contacted women enrolled in the study. The authors acknowledged that the sociodemographic characteristics of the sample could differ from those of the source group.

Despite all these potential benefits, only a few studies have used apps as experimental platforms; that is, mobile apps that are purposefully implemented to collect data, deliver experiment materials, and manage the random assignment across different experimental conditions, without the intervention of the researcher to mediate the interaction between the participant and the app. Typically, apps have been used to run the experimental condition paired with a non-app-based control condition, including, for instance, face-to-face training [22].

Mulcahy and colleagues [27] conducted a field experiment to test whether the level of sustainability of actual consumers could be improved by using game elements (including points, badges, and other rewards). To this end, they developed a custom app in collaboration with a local city government and were able to randomly recruit 601 real consumers. They showed that gamification positively affected consumers’ attitudes and behaviors toward bill savings.

A further study that was meant to improve sustainability-related behaviors employed a gamification app [28]. More specifically, they examined users’ experiences with a gamified app designed to promote sustainable energy behaviors. The results indicated that the sustainable behavior of turning off electricity switches could be encouraged through gamification. It should be noted that this study employed the “Reduce Your Juice” app, which was not purposefully developed to be an experiment platform. Indeed, the pre and post-test phases unfolded using different digital platforms.

Gamification apps have also been employed in longitudinal field studies. For instance, Feng and collaborators [29] analyzed a gamification app to investigate whether the type of game element, being commensurate and non-commensurate, had an impact on their effectiveness. The term commensurability refers to the extent to which consumers are able to quantify the value of a reward [30]. For example, commensurate game elements are directly associated with consumers’ performances, while incommensurate game elements are not related to their performances. The former were found to be associated with user performances, while the latter were connected to the satisfaction of psychological needs (i.e., autonomy, competence, and relatedness). However, it should be acknowledged that in this case, the apps were not differing only for the commensurate or non-commensurate game elements, but rather the entire app was different.

The study presented here aimed to overcome the above-mentioned limitations. More specifically, we purposefully developed a mobile app designed to be the experimental platform in which the participant should conduct all the experimental phases. In the present article, we report a field experiment that was meant to investigate the contribution of gamification features to foster compliance in a mobile application promoting physical activity.

## 2. Materials and Methods

### 2.1. The “Push up Game” Mobile App

“Push Up Game” (PUG) is a mobile app designed with the overall aim to help users perform the pushup exercise at home, and it is freely available on the main app stores. More specifically, the app is meant to keep track of the number of pushups completed by the user (the user is supposed to touch the screen with her/his nose to successfully complete and track one pushup). In the attempt to further help users not only to try out the app, but also to continue practicing over time, some gamification elements were included. In particular, gamification is exploited to foster a high level of user involvement, increase the time dedicated to workout, and promote higher retention rates. Therefore, the app was developed in three different versions. The “Counter version” included no gamification elements, and simply provided the user with the number of pushups she/he has performed. The “Buddy version” included three gamification features, which are Leaderboards, Levels of Achievement, and Social Pressure. Finally, the “Full version” included the same gamification elements as the “Buddy version” plus Digital Rewards and Challenges.

The users of the PUG mobile app (i.e., participants) were randomly and double-blinded divided into three groups, and each group was assigned a different condition associated with one version of the same app.

### 2.2. Game Design Elements

All the app versions had the same goal, the same interface, and graphic style and differed only in the features that were manipulated to be measured and tested. The game elements implemented in the different versions of the app were based on the work by Lister and colleagues [31], who, by reviewing the existing literature for the impact of gamification on health behavior, identified six core components of gamification. A detailed description of each app version is provided below (Table 1).

#### 2.2.1. Counter Version

**Figure 1:**
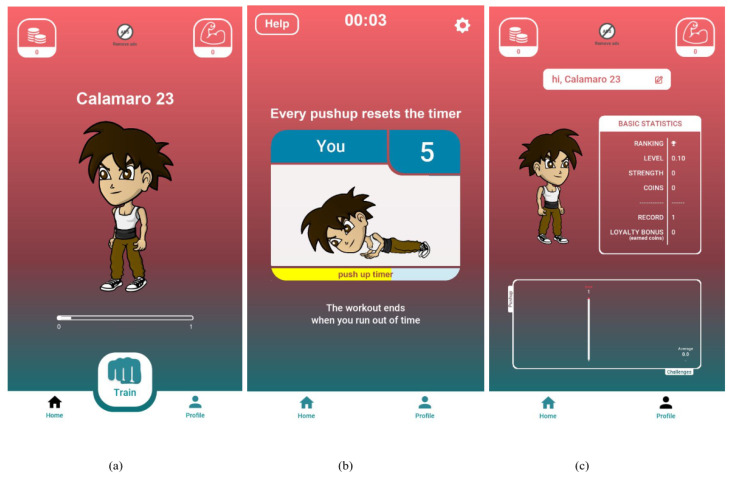
Screenshot of the app: Counter Version (C). (**a**) the homepage of the app, (**b**) a training session, (**c**) the profile section.

The main goal of the Counter Version (C version) is to enable the user to perform a basic training session, which consists of three sets of pushups. When the user starts training, the avatar is represented alone with a counter showing how many pushups the player is doing. This version has no gamification features (out of the six game elements identified by Lister and colleagues in their classification [31]), neither a target of pushups to be reached or specific goals to be achieved (Figure 1).

#### 2.2.2. Buddy Version

In the Buddy version (B version), the game elements of social or peer pressure, leaderboard, and levels of achievement are introduced.

**Figure 2:**
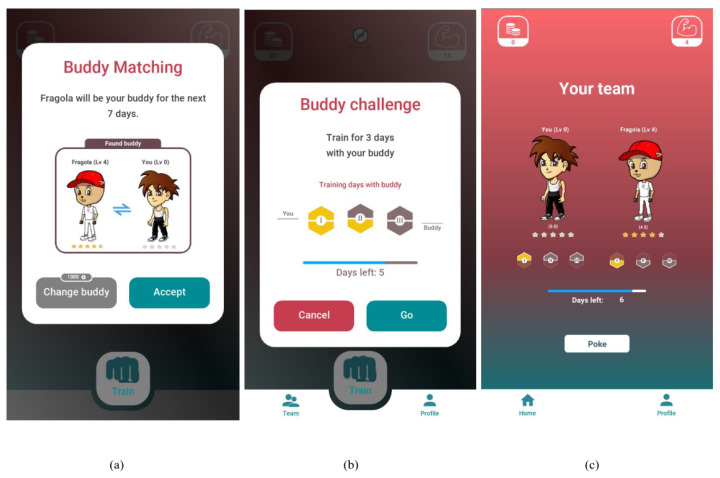
Screenshot of the app: Buddy Version (B). (**a**) the match with a new buddy, (**b**) the progress of the week goal, (**c**) the team section.

The goal of this version is to complete three challenges in a week. To accomplish these challenges, the user teams up with another random user, namely “the buddy”. Each of the three challenges must be completed by both the user and her/his buddy. Each challenge consists in completing one pushup session with no specific target. In this version, the goals to be achieved are limited to the number of training sessions completed, but there is no target to be reached at each session (Figure 2).

#### 2.2.3. Full Version

This version (F version) maintains the game elements present in the B version with additional competitions/challenges and digital rewards.

In this version, a challenge is composed of three sub-challenges, each represented by one minion, which is a little monster that in the games represents a servile and unimportant enemy that must be defeated. To defeat it, the user has to perform a calibrated minimum number of pushups (this number is personalized based on his/her average performance). As a result, the challenge ends up in a workout session composed of three sets of pushups (corresponding to the three minions to be defeated) and with a personalized number of repetitions (the number of pushups needed to defeat the minion).

A further novel element of this version is the “boss”, which is a virtual enemy that can be challenged only after having completed the three main challenges of the week. This feature recalls the game element of quests that in games are arduous tasks that players try to complete in order to gain a reward, and also introduces a more game-like and engaging dynamic for the user.

**Figure 3:**
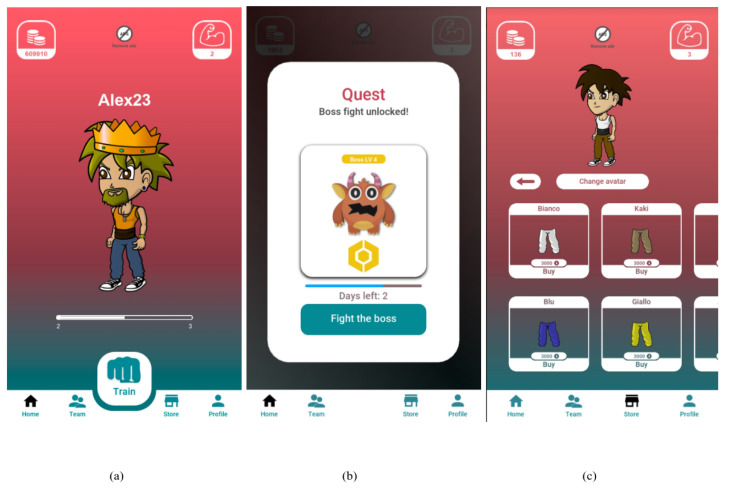
Screenshot of the app: Complete Version (F). (**a**) the app homepage, where at the bottom bar there is a shop icon, (**b**) the unlocking of the boss fight, (**c**) the shop section, where the user can exchange coins with virtual objects.

Finally, in this version, the user can gain coins by completing challenges, and they can also exchange these coins with virtual objects, called skins, to customize their avatar (Figure 3).

### 2.3. Experimental Design

The experiment followed a between-participant design. The between-participant factor has three levels, each corresponding to one version of the app.

We collected the following dependent variables:

- Kept days, which indicates the total number of days in which the user kept the app on her/his device, from the download day until the last day of app usage (data collection started on 9 June 2020 until 2 December 2021; for a total of 541 days);
- Workouts, which refers to the total number of workout sessions started by the user;
- Improvement, which indicates the difference between the user’s personal best performance and the pushup count recorded at the first workout session.

## 3. Sample and Data Description

### 3.1. Sample

The experiment involved a total of 13,245 participants with Android devices who spontaneously decided to download the PUG app. The participants provided their explicit consent to use the data collected by the app for scientific purposes. Their consent was collected using a digital consent form available within the app Right after the installation, the users were displayed an informed consent form. They had to provide their explicit consent to take part in the study and enable the data logging. Therefore, the data recording process started only for the users who accepted to participate in the experiment. As mentioned above, the assignment to the experimental group was randomly managed by the app.

Not all the users who downloaded the app effectively used it, as it emerged from the data logs. Therefore, before proceeding with the analysis, the data were pre-processed according to the following exclusion criteria:

- The minimum number of workouts recorded must be higher than 1;
- The record must be lower than 100. Some participants have values that are not compatible with pushups performed correctly. A cut-off of 100 was thus defined to exclude any unlikely performance (note that this filter applies to the best performance, so cheating even once would still result in removal);
- The number of pushups recorded in the first workout session must be greater than 1.

After the pre-processing, the final sample consisted of 832 participants.

### 3.2. Procedure

**Figure 4:**
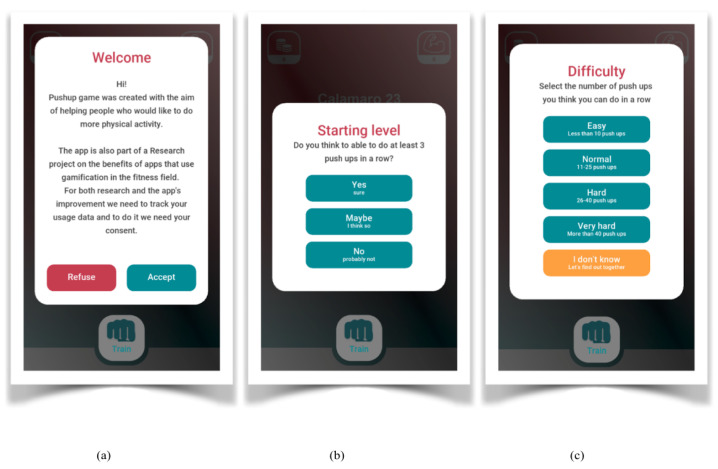
Screenshot of the app: Onboarding. (**a**) the terms and conditions allowing the data collection, (**b**) the question to establish the user’s expertise at performing pushups, (**c**) the question to set the level of difficulty of the training, asking the user how many pushups she/he is able to do.

At first access, an user ID is generated so as to anonymize the user. She/he is then randomly assigned to one of the three versions of the app. The first log was sent after the user clicked the “train” button for the first time. Every time the user would click on the “train” button, an anonymous CSV file was sent to the server that collected the data (Figure 4).

## 4. Analysis and Results

The analyses that are reported below refer to a comparison of the scores relating to kept days, workouts, and improvement between the three versions of the app to verify the effect of the game design elements (Section 4.1). Indeed, we compared the scores obtained by beginners and non-beginners users (sub-samples), and any relative differences for each version of the app, to test the effect of the users’ expertise in pushups (Section 4.2). Finally, a comparison of the scores between the three versions of the app considering the two different sub-samples was run to understand the effect of gamification on the level of expertise (Section 4.3).

### 4.1. Comparison of Different App Versions Regarding the Total Sample

The dependent variables were not distributed normally. More specifically, for the “Counter version”, the results of a series of Shapiro tests revealed that none of the variables were normally distributed: kept days (*W* = 0.52, *p* < 0.001); workouts (*W* = 0.59, *p* < 0.001); improvement (*W* = 0.72; *p* < 0.001). The same picture emerged for the “Buddy version” of the app: kept days (*W* = 0.59, *p* < 0.001); workouts (*W* = 0.47, *p* < 0.001) improvement (*W* = 0.79; *p* < 0.001); and for the “Full version”: kept days (*W* = 0.60, *p* < 0.001); workouts (*W* = 0.43, *p* < 0.001) improvement (*W* = 0.80; *p* < 0.001). Therefore, non-parametric tests were applied.

In particular, Kruskal–Wallis tests were carried out to verify the presence of significant differences between the versions, while Mann-Whitney tests were performed as post hoc tests for paired comparisons, as shown in Table 2. *p*-values were adjusted with Bonferroni correction [32] for multiple comparisons.

The Kruskal–Wallis test revealed a significant difference in the number of days in which participants in the three groups kept the application on their device ($X^{2}$ = 13.11, *df* = 2, *p* = 0.001). More specifically, Mann–Whitney tests (*W* = 63,795, *p* < 0.001) showed that users who had the Full version of the app (F version) kept it on their devices significantly longer (*Mdn* = 7) than those who had the Counter version (C version; *Mdn* = 5). No other significant differences emerged (Table 3).

The Kruskal–Wallis test also highlights a significant difference in the number of the started workouts ($X^{2}$ = 55.84, *df* = 2, *p* < 0.001). Paired comparisons showed that participants who installed the Full version of the app started significantly (*W* = 72,854, *p* < 0.001) more workouts (F version; *Mdn* = 6) than those who used the Counter app (C version; *Mdn* = 3) and more (*W* = 36,834, *p* < 0.001) than the Buddy version of the app (B version; *Mdn* = 4). While the difference between the Buddy version (B version; *Mdn* = 4) and the Counter version (C version; *Mdn* = 3) was not significant (Table 4).

The Kruskal–Wallis test indicates a significant difference in the participants’ improvement levels ($X^{2}$ = 7.06, *df* = 2, *p* = 0.029). More specifically, from the paired comparisons, a significant difference emerged (*W* = 48,978, *p* = 0.014) between the participants who used the Counter version of the app, who showed a greater improvement (*Mdn* = 10) than those using the Full version of the app (*Mdn* = 4). No other differences emerged (Table 5).

### 4.2. Comparison between Beginners and Non-Beginners in Pushups

The general sample was then split into two sub-groups, depending on the self-reported level of users’ expertise at the onboarding. More specifically, the users who reported being able to perform up to 10 pushups were grouped as beginners (N = 502). While all the users who reported being able to do more than 10 pushups were considered non-beginners (N = 330; Table 6).

The dependent variables were not distributed normally. More specifically, for Beginners, the results of a series of Shapiro tests showed that none of the variables were normally distributed: kept days (*W* = 0.42, *p* < 0.001); workouts (*W* = 0.51, *p* < 0.001); improvement (*W* = 0.75; *p* < 0.001). The following picture emerged for Non-beginners: kept days (*W* = 0.55, *p* < 0.001); workouts (*W* = 0.52, *p* < 0.001); improvement (*W* = 0.80; *p* < 0.001). Therefore, non-parametric tests were applied.

Mann–Whitney tests have been applied to investigate the differences between the two sub-groups about kept days, workouts, and improvement. Indeed, post hoc tests for paired comparisons have been run in the three different versions of the app to understand if the differences between the two sub-groups were also present within the versions of the app. Table 7 shows the descriptive statistics of the kept days, workouts, and improvement in the two groups of beginners and non-beginners (i.e., total) and in the two groups split by the app version.

The Mann–Whitney test highlights a significant difference in the number of days in which participants in the two sub-groups had kept the application on their device (*W* = 62,466, *p* < 0.001). More specifically, non-beginners kept it for a longer time (*Mdn* = 9) than beginners (*Mdn* = 5).

We further compared the two sub-groups of participants in each version of the app using Mann–Whitney tests. The analysis revealed (*W* = 8882, *p* < 0.001) that non-beginner users who installed the Full version of the app kept it on their personal devices longer (Non-Beginners; *Mdn* = 12) than beginner users (Beginners; *Mdn* = 5). Likewise (*W* = 5595, *p* = 0.006), non-beginners kept the Counter version of the app longer (*Mdn* = 8) than beginners (*Mdn* = 4). This finding suggests that non-beginners are more motivated to train and keep on despite the level of engagement of the app (Table 8).

The Mann–Whitney test highlights that also the number of started workouts between beginners and non-beginners is significant (*W* = 73,488, *p* = 0.005), with non-beginners (*Mdn* = 10) starting significantly more training sessions than beginners (*Mdn* = 5). More specifically, paired comparisons split by app version revealed that for the Full version of the app (F version), beginner users started significantly (*W* = 11,700, *p* < 0.001) more workouts (*Mdn* = 6) than non-beginner users (*Mdn* = 5), while (*W* = 6099, *p* = 0.004) for the Counter version (C version) non-beginner users started more workouts (*Mdn* = 5) than beginner users (*Mdn* = 3); (Table 9).

Notably, the Mann–Whitney test highlights that the difference in the improvement level between beginners (*Mdn* = 0) and non-beginners (*Mdn* = 0) is not significant (*W* = 77,168, *p* = 0.091).

### 4.3. Effect of Gamification Elements on the Level of Expertise

Finally, the effect of the gamification elements on the level of users’ expertise has been tested. Again, the variables were not distributed normally for beginners. More specifically, for beginners using the “Counter version”: kept days: *W* = 0.55, *p* < 0.001; workouts: *W* = 0.43, *p* < 0.001; Improvement: *W* = 0.82, *p* < 0.001). For beginners using the “Buddy version” of the app: kept days: *W* = 0.56, *p* < 0.001; workouts: *W* = 0.42, *p* < 0.001; Improvement: *W* = 0.72, *p* < 0.001). For beginners using the “Full version” of the app: kept days: *W* = 0.44, *p* < 0.001; workouts: *W* = 0.61, *p* < 0.001; Improvement: *W* = 0.63, *p* < 0.001).

The variables were not distributed normally also for non-beginners. More specifically, for non-beginners using the “Counter version”: kept days: *W* = 0.74, *p* < 0.001; workouts: *W* = 0.46, *p* < 0.001; Improvement: *W* = 0.73, *p* < 0.001). For non-beginners using the “Buddy version” of the app: kept days: *W* = 0.61, *p* < 0.001; workouts: *W* = 0.48, *p* < 0.001; Improvement: *W* = 0.83, *p* < 0.001). For non-beginners using the “Full version” of the app: kept days: *W* = 0.58, *p* < 0.001; workouts: *W* = 0.56, *p* < 0.001; Improvement: *W* = 0.80, *p* < 0.001). Therefore, non-parametric tests were applied.

#### 4.3.1. Beginners: Comparison of Different App Versions

No significant differences emerge in the number of days in which the beginners in each sub-group had kept the application in their devices ($X^{2}$ = 2.57, *df* = 2, *p* = 0.28), according to the Kruskal–Wallis test. However, a significant difference was shown for the number of workouts started by beginners ($X^{2}$ = 55.14, *df* = 2, *p* < 0.001).

Paired comparisons run using a Mann–Whitney test indicated (*W* = 27946, *p* < 0.001) that beginners who had the Full version of the app (F version) did more workouts (*Mdn* = 6) than those who had the Counter version (C version; *Mdn* = 3). In addition (*W* = 8742.5, *p* = 0.008), users who had the Buddy version of the app (B version) did more workouts (*Mdn* = 4) than those who had the Counter version (*Mdn* = 3). No other significant differences emerged (Table 10).

Furthermore a significant difference in the improvement level ($X^{2}$ = 14.29, *df* = 2, *p* < 0.001) emerged.

Paired comparisons indicated (*W* = 48,978, *p* = 0.014) that beginners who had the Counter version (C version; *Mdn* = 12) had a greater improvement than those who had the Full version of the app (F version; *Mdn* = 2.50). No other significant differences emerged (Table 11).

#### 4.3.2. Non-Beginners: Comparison of Different App Versions

The Kruskal–Wallis test did not highlight a significant difference in the number of days in the improvement level of non-beginners ($X^{2}$ = 0.87, *df* = 2, *p* = 0.646); but it did show a significant difference in the number of days in which non-beginners had kept the application in their device ($X^{2}$ = 8.31, *df* = 2, *p* = 0.016). Please refer to Table 6 for descriptive statistics.

Paired comparisons for kept days factor, using a Mann–Whitney test indicated (*W* = 9766.5, *p* = 0.007) that non-beginners who had the Full version of the app (F version) kept it on their devices significantly longer (*Mdn* = 12) than those who had the Buddy version (B version; *Mdn* = 6.50). No other significant differences emerged (Table 12).

The Kruskal–Wallis test did highlight a significant difference in the number of workouts started by non-beginners ($X^{2}$ = 11.02, *df* = 2, *p* = 0.004), with non-beginners using the Full version of the app starting more workouts (*Mdn* = 5) than those having the Buddy version (*Mdn* = 3; *W* = 10,050, *p* = 0.001). No other significant differences emerged (Table 13).

## 5. Discussion

Ecological validity and generalizability are relevant concerns for laboratory-based studies. This is especially true when it comes to investigating aspects related to human-computer interaction, which are intimately intertwined with the user’s motivation, attitudes, and more generally the context in which the system is deployed. Nowadays, personal devices, e.g., smartphones, can be exploited to collect data outside the laboratory, either using dedicated apps (e.g., the custom app developed by Mulcahy [27]) or relying on applications available on the market (e.g., “Walkup” and “Werun” used by Feng [29]). However, to the best of our knowledge, previous research required users to go to the laboratory or meet researchers at some point during the study, thereby disrupting the full realism of the experience. In the present study, we have investigated the effect of gamification elements embedded in a mobile app meant to increase the levels of physical activity. Three versions of the app were devised, each having different levels of gamification. The entire study was designed to run without the intervention of the experimenter, as such the app autonomously managed the random assignment to the different experimental conditions and the data collection.

Consistently with previous results [33], we found that game elements increased users’ levels of engagement, because participants using the version of the app featuring the highest level of gamification (namely the “Full version”) kept it on their smartphone longer and started more workouts. Interestingly, the level of improvement was higher for users who trained with the most basic version of the app, namely the “Counter version”, with no gamification elements, as compared to their counterparts using the Full version. This effect may be explained by referring to the different levels of interaction required by the gamification elements. Indeed, in the “Counter version” of the app participants only receive support for keeping track of the number of pushups they performed. On the contrary, in the “Full version”, additional functions were available, thereby grabbing at least some part of the users’ attention. Furthermore, we found that not all the patterns of gamification elements were equally effective for engaging participants. Indeed, participants assigned the “Buddy version” of the app, that is, the app featuring leaderboards, ranks, and social pressure, did not result in being more engaged than the users of the “Counter version” of the app.

Taken together, these findings suggest that gamification elements per se are not enough to increase users’ engagement or improve their performance, but rather a wise choice, and a combination of these elements should be made [34]. Moreover, it should also be noted that the gamification elements included did not affect users’ behavior to the same extent for expert and non-expert participants. Indeed, we found that users with higher self-reported expertise in performing pushups showed higher compliance, and were also more likely to start more workout sessions. This difference likely reflects the genuine different level of the users’ motivation. Future research should investigate the more appropriate pattern of gamification elements to better respond to the different levels of motivation of users.

The present work further contributes to the field by showing the feasibility of using only a mobile app for the full management of the experimental session, including the participants’ recruitment, random assignment to the experimental conditions, and data collection. While this method has the evident advantage of reducing the costs related to running the study, and more importantly, it enables the collection of genuine behavioral data, it also has limitations that should be carefully considered. Firstly, only individuals owning a mobile device and having at least some degree of digital literacy can participate, thereby resulting in a selection bias. Secondly, the fact that the app was freely available on the app store made it easy for every potential user to download it and give it a try. However, a large majority of them did not actually use the app. As a result, the actual data sample was much lower than the number of downloads. Finally, we have to acknowledge that our sample is a self-selected sample. This sampling method is largely employed and accepted, and it has the relevant advantage of reaching individuals who are genuinely motivated to get involved in the study. However, this comes at the cost of withdrawing the researchers’ control on the participants’ characteristics [35]. More specifically, we chose to collect very little information about participants’ background and experience of use, in an attempt not to disrupt the spontaneity of their everyday interaction with the app. While this choice was deliberately made to facilitate app retention, it came at the cost of missing a full understanding of participants’ background and actual experience with the app. As a consequence, we have to acknowledge that this limits the generalizability of our findings. Still, this point can serve as an indication for future research. Indeed, studies that intend to leverage mobile apps remotely and unobtrusively track spontaneous behavioral data, should also collect very detailed personal information, thereby enabling the possibility to control the homogeneity of the sample.

## 6. Conclusions

In the present work we showed that a mobile app can be profitably employed as an experimental platform to recruit participants, manage the random assignment to the experimental conditions, and collect naturally occurring behavioral data. Moreover, we manipulated the pattern of gamification elements embedded in the apps, and found that the combination of leaderboards, ranking, social pressure, rewards, and competition seemed to be the most effective to promote not only compliance, but also the amount of workout sessions. The app version featuring only some gamification elements did not seem to foster more compliance than the app with no gamification elements at all. Still, it should be acknowledged that our findings are to be interpreted cautiously due to the limited amount of information related to the sample Still, the present article contributes by demonstrating the feasibility of using mobile apps as self-managed experimental platforms.

## Figures and Tables

**Table 1:** Game elements included in each version of the APP, based on the list proposed by Lister and colleagues [31].

Game Element	Full Version	Buddy Version	Counter Version
Leaderboards	V	V	-
Levels of achievement or rank	V	V	-
Social or peer pressure	V	V	-
Digital rewards	V	-	-
Competitions/challenges	V	-	-
Real world prizes	-	-	-

**Table 2:** Descriptive statistics of the variables (i.e., Kept days, Workouts, Improvement) in the three experimental conditions (F, B, C version).

Factor	M	SD	MDN	IQR
	Kept days			
Total	20.02	39.44	6.00	17.25
F	27.81	53.42	7.00	22.00
B	15.33	24.46	6.00	12.75
C	13.25	20.58	5.00	12.00
	Workouts			
Total	7.42	10.34	4.00	5.00
F	9.76	12.61	6.00	8.00
B	6.53	9.26	4.00	3.00
C	5.11	6.67	3.00	4.00
	Improvement			
Total	18.22	25.43	7.00	27.25
F	15.44	25.16	4.00	20.00
B	18.69	23.85	10.00	29.00
C	21.29	26.28	10.00	33.15

**Table 3:** Post-hoc Mann–Whitney tests run on the whole sample of participants for the variable “Kept Days”.

Factor	Compared Conditions	*W*
Kept days	F, B	32,038
Kept days	F, C	63,795 *
Kept days	B, C	27,780

^*^ *p* < 0.016 (Bonferroni correction).

**Table 4:** Post-hoc Mann–Whitney tests results for the variable “Workouts” conducted on the whole sample.

Factor	Compared Conditions	W
Workouts	F, B	36,834 *
Workouts	F, C	72,854 *
Workouts	B, C	27,762

^*^ *p* < 0.016 (Bonferroni correction).

**Table 5:** Post-hoc Mann–Whitney tests results for the variable “Improvements”.

Factor	Compared Conditions	W
Improvement	F, C	48,978 *
Improvement	B, C	24,446
Improvement	F, B	27,218

^*^ *p* < 0.016 (Bonferroni correction).

**Table 6:** Size of participants’ sample split by version of the app used and self-reported level of expertise.

Version	Beginner	Non-Beginner	Total
F	200	163	363
B	66	100	166
C	236	67	303
Total	502	330	832

**Table 7:** Descriptive statistics (split groups). The table shows the descriptive statistics of the kept days, workouts, and improvement in the two groups (i.e., total) and in the two groups considering the different app versions.

	Beginner				Non-Beginner			
**Factor**	**M**	**SD**	**MDN**	**IQR**	**M**	**SD**	**MDN**	**IQR**
	Kept days							
Total	16.55	36.64	5.00	11.00	25.28	42.87	9.00	24.00
F	23.16	51.91	5.00	15.00	33.52	54.83	12.00	34.00
B	12.74	19.96	6.00	9.75	17.03	25.71	6.50	14.25
C	12.03	20.19	4.00	10.00	17.57	21.50	8.00	20.50
	Workouts							
Total	6.80	9.24	4.00	5.00	8.37	11.77	5.00	6.00
F	9.68	11.90	6.00	9.00	9.85	13.45	5.00	7.00
B	6.23	8.24	4.00	3.00	6.73	9.91	3.00	4.00
C	4.52	5.55	3.00	3.00	7.19	9.40	5.00	5.00
	Improvement							
Total	16.60	23.99	5.00	26.00	20.68	27.32	10.00	30.00
F	11.45	21.65	2.50	9.25	20.35	28.19	10.00	27.50
B	14.67	21.27	5.50	15.75	21.34	25.16	14.00	33.25
C	21.51	25.63	12.00	34.25	20.51	28.65	7.00	30.00

**Table 8:** Post-hoc Mann–Whitney tests results for the varaible “Kept Days” run on the sub-samples of beginners and non-beginners.

Factor	Compared Conditions	W
Kept days	Beginner, Non-Beginner in F	8882 *
Kept days	Beginner, Non-Beginner in B	2659
Kept days	Beginner, Non-Beginner in C	5595 *

^*^ *p* < 0.016 (Bonferroni correction).

**Table 9:** Post-hoc Mann–Whitney tests results for the variable “Workouts” conducted on the sub-samples of beginners and non-beginners.

Factor	Compared Conditions	W
Workouts	Beginner, Non-Beginner in F	11,700 *
Workouts	Beginner, Non-Beginner in B	2929.5
Workouts	Beginner, Non-Beginner in C	6099 *

^*^ *p* < 0.016 (Bonferroni correction).

**Table 10:** Post-hoc Mann–Whitney tests results for workouts in Beginners.

Factor	Compared Conditions	W
Workouts	F, B	6627.5
Workouts	B, C	8742.5 *
Workouts	F, C	27,946 *

^*^ *p* < 0.016 (Bonferroni correction).

**Table 11:** Post-hoc Mann–Whitney tests results for improvement in Beginners.

Factor	Compared Conditions	W
Improvement	F, B	24,446
Improvement	B, C	48,978 *
Improvement	F, C	27,218

^*^ *p* < 0.016 (Bonferroni correction).

**Table 12:** Post-hoc Mann–Whitney tests results for kept days in Non-Beginners.

Factor	Compared Conditions	W
Kept days	F, B	9766.5 *
Kept days	B, C	3174
Kept days	F, C	6293

^*^ *p* < 0.016 (Bonferroni correction).

**Table 13:** Post-hoc Mann–Whitney tests results for workouts in Non-Beginners.

Factor	Compared Conditions	W
Workouts	F, B	10,050 *
Workouts	B, C	2702
Workouts	F, C	5876

^*^ *p* < 0.016 (Bonferroni correction).
